# Towards an open grapevine information system

**DOI:** 10.1038/hortres.2016.56

**Published:** 2016-11-23

**Authors:** A-F Adam-Blondon, M Alaux, C Pommier, D Cantu, Z-M Cheng, GR Cramer, C Davies, S Delrot, L Deluc, G Di Gaspero, J Grimplet, A Fennell, JP Londo, P Kersey, F Mattivi, S Naithani, P Neveu, M Nikolski, M Pezzotti, BI Reisch, R Töpfer, MA Vivier, D Ware, H Quesneville

**Affiliations:** 1 URGI, UR1164 INRA, Université Paris-Saclay, Versailles 78026, France; 2 Department of Viticulture and Enology, University of California, Davis, CA 95616, USA; 3 Department of Plant Sciences, University of Tennessee, Knoxville, TN 37996, USA; 4 Department of Biochemistry and Molecular Biology, University of Nevada, Reno, NV 89557, USA; 5 CSIRO Agriculture and Food, Waite Campus, WIC West Building, PMB2, Glen Osmond, South Australia 5064, Australia; 6 Université de Bordeaux, ISVV, EGFV, UMR 1287, F-33140 Villenave d’Ornon, France; 7 Department of Botany and Plant Pathology, Oregon State University, Corvallis, OR 97331, USA; 8 Istituto di Genomica Applicata, Udine 33100, Italy; 9 Instituto de Ciencias de la Vid y del Vino (CSIC, Universidad de La Rioja, Gobierno de La Rioja), Logroño 26006, Spain; 10 Plant Science Department, South Dakota State University, BioSNTR, Brookings, SD 57007, USA; 11 United States Department of Agriculture-Agricultural Research Service-Grape Genetics Research Unit, Geneva, NY 14456, USA; 12 European Molecular Biology Laboratory, The European Bioinformatics Institute, Wellcome Trust Genome Campus, Hinxton, Cambridge CB10 1SD, UK; 13 Dipartimento Qualità Alimentare e Nutrizione, Centro Ricerca ed Innovazione Fondazione Edmund Mach, Via E. Mach 1, 38010 San Michele all'Adige, Italia; 14 UMR Mistea, INRA, Montpellier 34060, France; 15 University of Bordeaux, CBiB, Bordeaux 33000, France; 16 University of Bordeaux, CNRS/LaBRI, Talence 33405, France; 17 Department of Biotechnology, Università degli Studi di Verona, Verona 37134, Italy; 18 Horticulture Section, School of Integrative Plant Science, Cornell University, Geneva, NY 14456, USA; 19 JKI Institute for Grapevine Breeding Geilweilerhof, Siebeldingen 76833, Germany; 20 Department of Viticulture and Oenology, Institute for Wine Biotechnology, Stellenbosch University, Stellenbosch, Matieland 7602, South Africa; 21 Cold Spring Harbor Laboratory, Cold Spring Harbor, NY 11724, USA; 22 US Department of Agriculture-Agricultural Research Service, NEA Robert W. Holley Center for Agriculture and Health, Cornell University, Ithaca, NY 14853, USA

## Abstract

Viticulture, like other fields of agriculture, is currently facing important challenges that will be addressed only through sustained, dedicated and coordinated research. Although the methods used in biology have evolved tremendously in recent years and now involve the routine production of large data sets of varied nature, in many domains of study, including grapevine research, there is a need to improve the findability, accessibility, interoperability and reusability (FAIR-ness) of these data. Considering the heterogeneous nature of the data produced, the transnational nature of the scientific community and the experience gained elsewhere, we have formed an open working group, in the framework of the International Grapevine Genome Program (www.vitaceae.org), to construct a coordinated federation of information systems holding grapevine data distributed around the world, providing an integrated set of interfaces supporting advanced data modeling, rich semantic integration and the next generation of data mining tools. To achieve this goal, it will be critical to develop, implement and adopt appropriate standards for data annotation and formatting. The development of this system, the GrapeIS, linking genotypes to phenotypes, and scientific research to agronomical and oeneological data, should provide new insights into grape biology, and allow the development of new varieties to meet the challenges of biotic and abiotic stress, environmental change, and consumer demand.

## Introduction

Grapevine is a perennial plant that has been cultivated for more than 7000 years in many environments and according to many different viticultural practices. It is a globally important crop, eaten fresh or processed into various products including wine (http://faostat3.fao.org/). Like other crops, it faces changing biotic and abiotic stresses linked to climate change or the introduction of exotic pests (see for instance Duchene *et al.,*^1^ Hannah *et al.*^2^ and van Leeuwen *et al.*^3^). The grape and wine industries, must in addition, cope with societal demands to reduce environmental impacts (for example, by reducing phytochemical treatments) and improve product safety (for example, reducing chemical residues in products) while maintaining cost-effective and sustainable production. Thus, the major challenges for viticulture and enology (and the primary focus of research) are to control the final berry composition at vintage in variable environments and to sustain yield and quality while limiting the use of pesticides, water and other inputs.

In order to address the scientific questions related to these challenges, the grapevine research community is increasingly using high-throughput data-generative experimental techniques (‘omics’ technologies) that generate large and heterogeneous data sets describing genotypes, phenotypes (transcriptome, proteome, metabolome, phenome, development stages, mutant or extreme phenotypes and so on) and the environment. Indeed, during the last 15 years, several high-throughput data sets from grapevine have been published, including Expressed Sequenced Tags (ESTs) (for example, Da Silva *et al.*^4^), simple sequence repeats (SSRs) and single-nucleotide polymorphisms (SNPs) molecular markers (for example, Bowers *et al.,*^5^ Pindo *et al.,*^6^ Myles *et al.*^7^), QTL maps (for example, illustrating two very different kind of traits^8,9^) and transcriptomes (for example, among many others^10–12^). The determination of the genome sequence of grapevine in 2007^13^ created new possibilities for transcriptomic and proteomic studies (for example, among many others^14–16^) and for better describing and understanding genome grapevine genetic diversity either through genotyping/re-sequencing studies or *de novo* sequencing of new genotypes.^7,17–19^ Phenotypes of different nature have been studied (often in studies aimed at associating phenotypic changes with genetic variations) and here too, throughput has notably increased in recent years: for example, the study of single metabolites has been increasingly replaced by metabolomics studies (for example, Zamboni *et al.,*^14^ Doligez *et al.*^20^ and Fournier-Level *et al.*^21^) and manual field or greenhouse scoring by the use of more automated processes (for example, Marguerit *et al.*^9^ and Coupel-Ledru *et al.*^22^).

The greatest value of these data sets depends on their integration to generate new knowledge, and therefore on the ability to combine the results of different experiments. To allow this, data should be Findable, Accessible, Interoperable and Reusable (FAIR principles^23^). An emblematic model in the plant community is *Arabidopsis thaliana*, for which rich data sets are available and which has been used to derive working hypotheses for gene function in crop species. This has been supported by the TAIR portal (www.arabidopsis.org) and the more recent Arabidopsis Information Portal (www.araport.org). However, in grapevine, the increasing wealth of data is highly dispersed and often poorly accessible, hindering its effective exploitation beyond the scope of its initial production. Moreover, in the absence of dedicated funding and sufficient international collaboration, there is no information portal targeted at the grapevine research community. Although large international repositories do exist for molecular biological data (for example, the European Nucleotide Archive, GenBank), these do not systematically capture the detailed knowledge related to genome function (for example, regulation networks, metabolic networks), the plant material used and any non-molecular phenotyping data that is the specific expertise of grape researchers. Instead, these data are at best published along with research papers and managed in regional and local databases, or at worst isolated on individual researcher’s computers and completely inaccessible to the wider community. There is a clear need for research policies that create incentives favoring data sharing to improve the quality of research results and foster scientific progress.^24^

The interpretation of previously published data always requires additional ‘metadata’ to provide the appropriate context. In addition, both data and meta-data should also be formatted in standardized representations to enable its processing in an automated manner and avoid errors generated by manual manipulations, especially in the case of very large data sets.^23^ This requires community-wide agreement on guidelines for annotation, tools for data preparation, and the dedicated custodianship of important/exemplar data. Although generic solutions exist for many data types individually, much grapevine data is still far from FAIR, and little support is available for community members to make it so.

**Figure 1:**
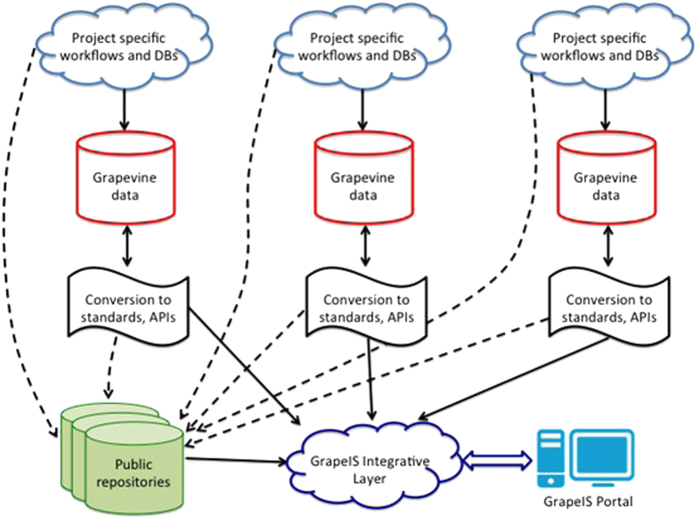
Conceptual scheme of the grapevine distributed information system (GrapeIS).

In 2014, in response to the demands of the grapevine research community, the International Grapevine Genome Program (IGGP; www.vitaceae.org) consortium launched an action to define a strategy for the stewardship of grapevine genomic data to allow their easy access and reuse. The first output was the proposition of a gene nomenclature;^25^ the second expected output is a strategy for the broader management of diverse grape data in accordance with the FAIR principles. In this paper, we outline such a strategy for the development of a global Grape Information System (GrapeIS, http://www.vitaceae.org/index.php/Bioinformatics), a platform to enable access (by humans and machines alike) to a broad collection of data sets and reference data from a wide variety of sources with a flexibility that promotes the rapid introduction of new data sources derived from new and emerging technologies. To meet these objectives, we have devised a plan inspired in part by the experiences of the international WheatIS initiative that provides a portal for wheat data (http://www.wheatis.org/) and by the transPLANT infrastructure for plant genomic science (www.transplantdb.eu) that allows data integration from nine distinct European databases. The GrapeIS will comprise an open federation of independent information systems (nodes) interconnected by a central web portal (Figure 1), and will provide a toolset to reduce the costs of data publication and interrogation. This will provide a robust, cost-effective model for data integration by exploiting the expertise of existing resources, and best practice and data standards from related research communities grappling with similar problems.

## Review and discussion

### Discovering data stored in distinct databases from a single entry point: interoperability of the infrastructures

One model for providing integrated access to diverse data sources features a single data custodian, who takes comprehensive responsibility for the storage and integration of all relevant data. An alternative model is to provide an integrated query engine providing a common entry point to dispersed resources, each of which might contain different data (and have a different focus of interest). The second model has the advantage of exploiting (rather than replacing) existing resources (and their sources of funding). Such a common entry point should (i) allow the discovery of different data types (for example, omics data, phenotypic data, climatic data) or data sets of the same type (for example, multiple genome re-sequencing projects), (ii) facilitate their integration (for example, a catalog of all the genotypic and phenotypic evaluation data known for a given set of varieties) and (iii) facilitate the import of these data into diverse analysis or visualization tools. Achieving this requires a commitment from all contributing resources to serving data in accordance with a set of common standards, such that it can be automatically interrogated in a standard way.

The first step in providing FAIR data is ‘findability’. A model for findability for plant-focused resources has been established by the transPLANT project. The transPLANT integrated search engine^26^ operates using the generic SolR (http://lucene.apache.org/solr) search engine to provide search facilities over remote data files published by each participating resource conforming to a minimal standard schema (which allows for a faceted search to be provided, giving users the options to winnow large results sets based on commonly useful criteria). Access is provided through a common search portal and via RESTful web services.

To support more advanced knowledge extraction, the automatic manipulation of data sets, and the efficient and correct re-analysis and re-use of data, a more advanced model is required.^27^ Data needs to be annotated with detailed and accurate metadata, requiring both manual curation and automated quality control (these tasks can be distributed or centralized, but are needed regardless of whether a resource is centralized or federated). Where multiple resources are collaborating, agreement on a common set of controlled vocabularies is required; if vocabulary terms are structured as ontologies (with the definition of clear semantic relationships between the terms), the power of potential queries is increased. In developing such a model, the grape community will be able to draw on other ongoing efforts. Moreover, standard formats must be agreed for publishing such data; and appropriate forums identified for publicizing its availability.

Standard formats already exist for many types of data: for example, General Feature Format (GFF3; http://gmod.org/wiki/GFF3) and Genbank (GBK; https://www.ncbi.nlm.nih.gov/genbank/samplerecord/) for genome and aligned data, Variant Call Format (VCF; http://vcftools.sourceforge.net/specs.html) for nucleotide sequence variants, Binary Alignment Format (BAM; http://www.htslib.org/) for next-generation sequence alignments, BioPAX (www.biopax.org) and Systems Biology Mark-up Language (SBML; http://sbml.org) for pathways and networks, PSI-MI XML standard for proteomic data (http://www.psidev.info/node/60#mi-purpose)^28^ and a suite of standards are being proposed by the Data Standards and Metabolite Identification Task Groups of the international Metabolomics Society for metabolites analysis (http://www.metabolomics-msi.org/),^29^ as in untargeted metabolomics, robust and standardized structural annotation of metabolites appears crucial to maximize their interpretation and impact.

Moreover, international initiatives are on-going to agree on data models that specify APIs for different types of data in relation to plant breeding (genotypes, phenotypes, markers and so on; http://docs.brapi.apiary.io/#), genomics (expression, variation and so on; https://genomicsandhealth.org/work-products-demonstration-projects/genomics-api; https://dpb.carnegiescience.edu/labs/huala-lab/projects/plant-genomics-interface-plain),^30^ and with any other specific purpose (for example, for phylogenetic studies in Ayres *et al.*^31^). Other initiatives as for instance BioSharing (https://biosharing.org/), exist to publicize resources with a commitment to providing open data.

With limited resources, a sensible strategy for the grapevine community is to promote the use of existing international repositories for common data types (for example, European Variation Archive, EBI Gene Expression Atlas, the Gene Expression Omnibus (GEO), MetaboLights, PRIDE and so on), which already require submission of standards-compliant data, and to utilize these data (alongside other grape-specific data) in specialized services targeted at the specific needs of grapevine researchers. This has been the strategy of the grapevine community from its start regarding molecular data (sequences, polymorphisms, proteomics, metabolomics). For instance, 3971 grapevine transcriptomic data sets have been so far submitted to the GEO database (for example, Moretto *et al.*^32^). In turn, phenotypic data are not currently concentrated in any generic resource, nor is there an obvious repository to which submission can be recommended. The grapevine community must therefore assist in the coordination of multiple resources and should contribute to the definition of international standards in the domain. As many of the data will have features in common with those produced by other crop communities, coordination with wider initiatives such as the European Plant Phenotyping Infrastructure (EMPHASIS, http://www.plant-phenotyping.org) is a sensible course.

### Capturing the data of the grapevine community in standard formats: toward data interoperability

**Figure 2:**
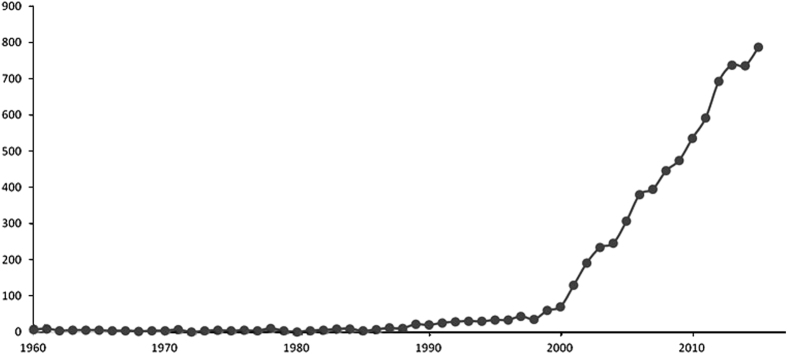
Evolution of the number of published papers retrieved from the PubMed database (http://www.ncbi.nlm.nih.gov/pubmed) between 1960 and 2015 with the query ‘grapevine’ OR ‘Vitis’.

Looking backward, the grapevine community has been increasingly active in the production of data in the life science area, as shown by a very naive search of recent publications (using query terms ‘grapevine’ OR ‘vitis’) in the PubMed database (Figure 2). The data described in the papers are very diverse covering genomes, genotypes, genomic variation, genetic maps, QTLs, association genetics, transcriptomics, proteomics, metabolomics, phenotype characterizations; and rapidly developing, with the quantity of data produced by a single experiment increasing rapidly over time. The development of a common policy for data standardization has lagged and this gap is impairing progress in grapevine research.

### Minimal information about experiments

The foundation of data sharing is to have a good understanding of what is about to be shared. For certain common types of experiments (and particularly for experimental techniques), agreement should be possible about the information that needs to be provided alongside the experimental results in order for that data to be useful and interpretable by others. This idea has been captured, for many experimental types, in ‘Minimum Information’ papers, in which the conceptual metadata needed to support an experiment of that type are defined. Among the metadata standards that might be of interest for the grapevine community are already in common use, including the Minimal Information About a Microarray Experiment (MIAME),^33^ now evolving into the Minimal Information about high-throughput SEQuencing experiments (MINSEQ, http://fged.org/projects/minseqe/) and the Minimal Information About Proteomic Experiments (MIAPE),^34^ the Metabolomic Standards Initiative has developed a standard for Core Information for Metabolomics Reporting.^35^ Such papers have formed the basis for the subsequent development of exchange formats and databases. Others standards are still emerging like the Minimal Information for QTLs and Association Studies (MIQAS, http://mibbi.sf.net/projects/MIQAS.shtml), the Minimal Information about a Genotyping experiment (MIGen, http://migen.sourceforge.net/) or the Minimal Information About Plant Phenotyping Experiments^36^ (MIAPPE, http://www.miappe.org/). Experimental metadata within-omics experiments can be conveniently standardized and shared with the ISA-Tab protocols.^37^ The success of these standards obviously depends on their adoption by the community, which is determined by many factors, such as its enforcement by publishers and the existence and ease-of-use of an associated toolset.^38^ Widespread adoption requires that correct formatting of data must be as simple as possible. On the other hand, if time consuming development of specific tools is required, there is a risk that a format will be slow to evolve, and at risk of being desynchronized with the needs of the data producers in a period where technologies are evolving very rapidly.^38^

### Plant material identification

Inevitably, the understanding of processes that underlie sustainable crop production under varying environmental conditions requires experimentation with a wide diversity of genetic material. This could include the use of mutants or individuals carrying extreme phenotypes to decipher physiological mechanisms, progenies derived from controlled crosses or diversity panels to determine the genetic control of trait variation, individuals collected *in situ* for the study of the adaptation of populations to environments, the evaluation of wild relatives and so on. In the grapevine community association studies, exploiting natural diversity through large-scale sequencing and phenotyping, have enormous potential to compensate for the lack of large mutant collections and are widely implemented to complement other approaches to support the identification of candidate genes for traits in physiological processes (for example, Fournier-Level *et al.,*^21^ Nicolas *et al.*^39^). Importantly, many studies not only involve diverse genotypes of *Vitis vinifera* (the most widely cultivated species), but also related wild species, which are especially interesting in the context of improving tolerance to biotic and abiotic stresses (for example, Venuti *et al.*^40^). The ability to integrate such data from different laboratories thus first of all relies on the correct and unambiguous identification of the plant material used, a problem shared by many crop communities. It is of high importance that data always contain an unambiguous identification of the species, cultivar/variety and the accession from which the studied sample was derived.

International coordination in this regard has been ongoing since the mid-seventies. The FAO/Biodiversity Multicrop Passport Descriptors^41^ (MCPD; http://www.bioversityinternational.org/e-library/publications/descriptors/) is widely recognized as the metadata standard for crop genetic resources (http://www.bioversityinternational.org/e-library/publications/detail/faobioversity-multi-crop-passport-descriptors-v2-mcpd-v2/), and has been adopted by the curators of germplasm repositories and implemented in their information systems. In these, for a given crop, a pair value corresponding to the accession number and the genebank or laboratory holding it defines the entities (that is, a plant) to which accession-specific information is assigned. For example, several accessions of the Cabernet Sauvignon cultivar are maintained in different gene banks of the world, clearly identifiable by the combination of their holding institute and their accession numbers (see the European *Vitis* Database www.eu-vitis.de, EURISCO eurisco.ipk-gatersleben.de/ or GRIN www.ars-grin.gov/npgs/index.html databases). Some years ago, the plant genetic resources community has proposed to associate to each accession an international Permanent Unique IDentifier (PUID). Recently, in support of this effort, guidelines, a dedicated infrastructure and a revision of the MCPD (v2.1) have been set up by the International Treaty on Plant Genetic Resources for Food and Agriculture to provide genebanks with these PUIDs (http://www.fao.org/plant-treaty/areas-of-work/global-information-system/doi/en/). However, PUIDs are not yet used for the identification of grapevine accessions. Moreover, the information needed for the unambiguous identification of accessions is often poorly linked to experimental data sets derived from these materials.

In vegetatively propagated perennial species such as grapevine, clonal variation, history, languages, misspelling and mis-identification in germplasm collections can lead to situations where different genotypes share a common cultivar name (for example, for ‘Augusta’ in Table 1) or conversely the same genotype has different cultivar denominations (for example, for ‘Cabernet franc’ in Table 1). In addition to the development of a unique identification system of accessions, the European grapevine repositories have therefore also agreed on an unambiguous identifier for cultivar names to tackle the problems of synonymy and homonymy. This cultivar identifier is currently maintained by the *Vitis* International Variety Catalog (*V*IVC, www.vivc.de) and yet very poorly used in published data sets although it could greatly improve their reusability.

Laboratories often develop their own identification system for plant material (cultivars, accessions and derived samples) maintained at their own sites, rather than in coordination with germplasm repositories. The origin of a plant material, whether from a repository or a laboratory, is therefore a mandatory information within any minimal information delivered along with data sets, to avoid confusion in the identification of the plant material. These various identifiers are often poorly used and described in submissions to archives of molecular data, making it hard to cross-reference molecular data and individual materials.

### Controlled vocabularies/ontologies

The use of ontologies, in which controlled terms are integrated using hierarchical semantic concepts, allows the integration of data sets where information has been captured at different levels of granularity. Depending on the variety of the relationships utilized, more complex semantic reasoning and potential discovery of emergent properties can also be envisioned. A good example of the use of ontologies for crop data is the work coordinated by Bioversity International (http://www.bioversityinternational.org/) which in 1976 started to develop crop-specific controlled vocabularies for a limited number of traits allowing germplasm identification, and which subsequently has aimed to develop comprehensive and detailed dictionaries of controlled vocabularies for germplasm description^41^ and to transform these into crop-specific ontologies (http://www.cropontology.org/). A major aim is to standardize the descriptions of the measured variables (target trait, unit, protocol), which is mandatory for consistent comparisons of data sets from different origins. A current focus is to complete these for traits related to breeding projects. More generic ontologies exist for many other types of biological descriptors (for example, the Plant Ontology, which describes plant anatomy,^42^ or the Gene Ontology,^43^ which describes gene function).

However, if data formats are generic, model system ontologies cannot always be directly applied to grapevine data as the botanical family significantly diverges from ‘model’ species in a number of crucial ways: grapevine is a perennial liana mostly cultivated through grafting, with different genotypes for their rootstocks and scions, each highly heterozygous. In many aspects, wine grapes more resemble other crops used as luxury crops (for example, tea, coffee, cocoa and so on), where the phenotype related to the quality of the final product greatly prevails over the growing plant phenotype and yield. As a consequence, the relationship between the chemical composition and morphological phenotype of the berry and the quality of the resulting wine adds further complexity in the data to be integrated to address questions of interest for the crop. Recently, a new grape-specific ontology has been developed to capture traits (from plant phenotyping to wine-related data) and the experimental conditions under which those traits are measured (http://www.cropontology.org/ontology/VITIS/Vitis). This has been built from descriptors developed by the International Organization of Vine and Wine (www.oiv.int) and based upon grapevine standards widely used by the grape community since the 1980s, and its widespread adoption is likely to be critical for the success of the GrapeIS.

### Genome structure, genome expression and genome variation

Many biological data types can be expressed with respect to locations on genomic sequence, allowing that sequence to function as a focal point for the integration of data. Among the most important of these to the grapevine community are genes and genetic markers that are key concepts for genetic and genomic studies and, as a consequence, for data interoperability in plant biology. Comprehensive, regularly updated and curated catalogs of grapevine genes and markers would therefore be a very useful tool for the grapevine community.

A nomenclature for grapevine genes has recently been published,^24^ but the scientific tools enabling gene identification and characterization, which include new and improved genome sequences, annotation protocols, and methods for functional characterization, are still evolving. Standardization description of gene function and interactions (pathways and networks) is of critical importance to allow the integration of state-of-the-art knowledge from multiple sources. The extent of standardization varies according to data type: for example, data for gene expression is better standardized in databases such as GEO (http://www.ncbi.nlm.nih.gov/geo/) than for proteins or metabolites. For metabolite data, the discrepancies within compound structures, purification protocols, and analysis methods make standardization an especially difficult problem. In recent years, some new resources supporting standardized metabolite data such as MetaboLights (http://www.ebi.ac.uk/metabolights/) have been emerging. Another interesting effort is The Metabolomics Workbench^44^ (http://metabolomicsworkbench.org/) that aims at delivering a public repository for metabolomics metadata and experimental data spanning various species and experimental platforms, metabolite standards, metabolite structures, protocols, tutorials and training material. In parallel, a grapevine-specific metabolic pathway database was developed using hierarchical schema based on gene ontology and enzyme function (VitisCYC^45^). But these efforts need to be more widely promoted within the grapevine community as only five experiments from two laboratories and related to *Vitis vinifera* have been deposited so far in MetaboLights (two related to living tissues and three from wine extracts).

In turn, the PRIDE archive (https://www.ebi.ac.uk/pride/archive/) is the most recognized proteomics database. Another specific database exists for protein data, PhosphoSitePlus^46^ (PSP http://www.phosphosite.org/homeAction.action), fulfiling a complementary role from PRIDE. PhosphoSitePlus is an online resource providing comprehensive information and tools for the study of protein post-translational modifications including phosphorylation, ubiquitination, acetylation and methylation.^46^ So far, there are 10 grapevine experiments published in the database, which is encouraging in terms of openness of the data given that fewer proteomics than metabolomics experiments are carried out: a search in PubMed with the keywords (grapevine AND (Vitis)) OR Proteom* gather 138 papers from the literature, while the keys words (grapevine AND (Vitis)) OR Metaboli* gather 3270 papers.

With genetic marker data, there are similar challenges to those of genes: synonymy, homonymy, the necessity to evolve the linked information in relation with new genomes and new genome versions and in addition, the use of novel increasingly high-throughput technologies. Data that should be captured include the technology that was used for their identification, the initial genetic material from which they were derived and their position on a reference sequence. There are possible standards that could be adopted to handle this data type, including the Minimal Information about any (x) Sequence (MIxS, http://wiki.gensc.org/index.php?title=MIxS), and the Molecular Marker Ontology developed under the umbrella of Bioversity International (http://www.cropontology.org/ontology/CO_500/Molecular%20marker). So far, most of the currently used markers have been archived at NCBI (dbSNP and dbVAR databases) under early IGGP recommendations. EMBL and NCBI archives are an important sources of recommendations for data standardization in this quickly evolving field.

**Figure 3:**
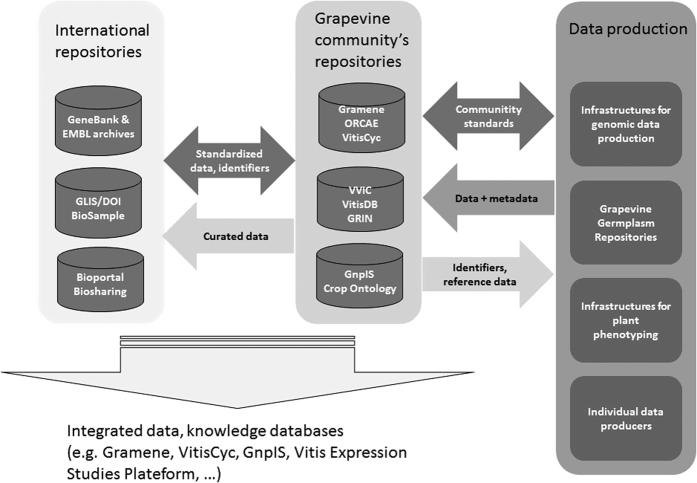
Different categories of infrastructures that should contribute to the GrapeIS and their key relationships. Within each category, the list of infrastructures cited is not exhaustive but rather meant to be an illustration of its possible content.

Based on the present review of the practices and possibilities in terms of data management for grapevine, Figure 3 describes different categories of participants that could contribute to a GrapeIS, and the key relationships between them. The first category of participants are data producers, involved in nucleotide sequencing, metabolomics, proteomics, and phenotyping (increasingly using high-throughput platforms), germplasm repositories and individual laboratories. It is the responsibility of these groups to publish well-formatted data sets with complete metadata and well described measured variables to the second category of contributors, the data repositories. These vary from generically focused, international efforts (for example, Genesys for genetic resources, EMBL and NCBI archives for various genomic data, see Figure 3) to smaller, community-maintained repositories, focused on grapevine-specific problems or national datasets^32,45,47–50^ (Figure 3).

### Conclusions

The policies of research agencies all across the world are increasingly enforcing measures aiming at improving the FAIRness of public data based on the statement that sharing precompetitive data is a strong fuel for new discoveries but also for innovations. Indeed, only FAIR data can be easily found by virtually any kind of users and re-used, including in combination with private data.

There are several components to be implemented by an initiative such as the GrapeIS to increase significantly the FAIRness of the public data produced by the grapevine research community. First, the GrapeIS has to be developed in the frame of an international consortium aiming at representing the whole community. This will include setting up the necessary networking activities including a platform for discussing the roadmaps to support the development of the GrapeIS and to follow up needs. A first step has been achieved with the writing of the present paper, authored by members of the IGGP steering committee and domain experts representing 9 countries and 18 public institutes. Still, the challenge will be to sustain the initiative through funding mechanisms such as the Research Coordination Networks of the National Science Foundation (USA) or COST Action (EU) for the networking activities and the writing of various aligned collaborative projects to implement or develop dedicated tools and software, produce large curated data sets and so on. Ideally, the implementation of common and clear guidelines toward FAIR data in all the projects developed by the grapevine community, which is, moreover, more and more required by the funding agencies, would already create a favorable ground for the implementation of any distributed information system.

Among its first activities to be developed, the initiative needs therefore to firmly re-advocate the submission of standard data to established repositories with regularly updated recommendations and guidelines. These repositories would provide a persistent home for submitted data, and stable identifiers associated with these and well designed in collaboration with the data producers, to allow its retrieval and integration. Other key roles for repositories include coordination of data producers and consumers in the development of standards, the development of data validation and submission tools to reduce the cost of standards-compliance challenge,^38^ the development of analysis tools focused on user problems, the maintenance of high-quality documentation and the development of training programs to spread good practices regarding data management and analysis. Indeed, it is in the interest of the crop communities to support data sharing and re-use by setting up working groups playing an active role in the development, validation and dissemination of recommendations and tools for data description, formatting, archiving and publication. These working groups acting in the frame of the IGGP activities on data standardization represent a very important component of the GrapeIS initiative and would help their communities of data producers to use the commonly adopted formats and to keep pace with evolutions in the domain.^28,29^

Repositories require stable funding (or at least, a transition plan to ensure the safeguarding of their data should funding cease). Often funding schemes are temporary, making it hard for repositories to make sound long-term plans. Coordination of Europe’s biological data repositories is now being led by the ELIXIR life sciences infrastructure (https://www.elixir-europe.org), which is exploring how to make such resources more sustainable. This is still a difficult challenge, but the use of open standards facilitates the development of softwares by the wider community. If these softwares are also published under open-source licenses, common solutions could emerge that could be adopted by many different repositories, working on grapevine but also for other crops or organisms, reducing the cost compared to a system where every group independently develops a complete, proprietary software stack. In this paper, we have proposed a new resource, the GrapeIS, designed to provide integrated access to diverse infrastructures providing grapevine data, with some guaranties of sustainability of the whole system: the federation of infrastructures, the use of open common standards and the animation and dissemination by the IGGP international consortium.

The last important component for the design of a FAIR compliant sustainable information system will be that it is useful to a large group of diverse users. Like the data producers, users also have an important contribution to make in specifying the data models, the goals of the repositories and of the whole GrapeIS infrastructure. Data users can be very diverse and the priority of the IGGP are the researchers in the field of plant biology in public institutions (which also are the main producers of public data) or in private companies, breeders from the public and the private sector, engineers from extension services for grape and wine production, teachers and students. Some data can also be of interest for growers or for the general public (for example, the catalogs of germplasm collections) and the GrapeIS initiative might in time help as well to transfer more of the knowledge produced by the scientific community to a broader public. Again, the IGGP international consortium will have an important role in organizing two-way interactions between all the stakeholders of the initiative: users, partners building the GrapeIS and funding agencies.

## Figures and Tables

**Table 1:** Synonymy, homonymy, clonal variation, history, languages, misspelling and misnaming contribute to confusing accession names across collections and studies

*Variety prime name*^a^	*Variety number*^a^	*Accession name*^b^	*Accession code*^b^	*Taxon*^c^	*Country*^c^
AUGUSTA	771			*Vitis vinifera* L. subsp. *vinifera*	ITA
	772			*Vitis labrusca* L.	CAN
	773			*Vitis labrusca* L.	USA
	14 781			*Vitis vinifera* L.subsp. *vinifera*	ROU
	21 288			Interspecific cross	HUN
CABERNET FRANC	1927	Cabernet franc	324Mtp1	*Vitis vinifera* subsp *vinifera* cv. Cabernet franc	FRA
		Cabernet franc no. 23	324Mtp14		
		Breton no. 3	324Mtp25		
		Gros Bouchy	324Mtp37		
		Cabernet franc no. 1	324Mtp38		
		Cabernet franc no. 2	324Mtp39		
		Cabernet franc	324Mtp43		
		Cabernet franc 1	324Mtp44		—
		Crouchen negre=Morenoa	324Mtp47		
		Hartling	324Mtp48		CZE
		Cabernet no. 9	324Mtp5		—
		Odjalechi noir (par erreur)	324Mtp50		
		Chenin noir	324Mtp51		HUN
		Cabernet no. 13	324Mtp6		FRA
		Cabernet no. 17	324Mtp9		FRA

The *VIVC* catalog proposes a most frequent variety name (the ‘prime name’). However, the only unambiguous way for tagging a variety is the ‘variety number’ given by *V*IVC. For instance, the variety ‘Augusta’ is described five times in the *V*IVC database originating from different countries. Each of these entries corresponds to a different genotype and sometimes different species. Another example of the possible difficulties arising from accession names is illustrated below with the accessions corresponding to ‘Cabernet franc’ in the Vassal collection as retrieved from the GnpIS-Siregal portal of the germplasm collections maintained by the French National Institute for Agronomical Research (INRA).

a From the *V*IVC database (www.vivc.de).

b From GnpIS-Siregal (https://urgi.versailles.inra.fr/siregal/).

c From GnpIS-Siregal for Cabernet franc and from VIVC for Augusta.
